# An Effect of Layered Auxiliary Cathode on Thickness Uniformity in Micro Electroforming Process

**DOI:** 10.3390/mi14071307

**Published:** 2023-06-25

**Authors:** Huan Wang, Jianpeng Xing, Tao Fan, Jinhu Liu, Jing Xie, Chaobo Li

**Affiliations:** 1 Institute of Microelectronics of the Chinese Academy of Sciences, Beijing 100029, China; 2 University of Chinese Academy of Sciences, Beijing 100049, China

**Keywords:** micro-electroforming, thickness uniformity, auxiliary electrode, edge effect

## Abstract

Thickness nonuniformity is a bottleneck in the micro electroforming process of micro-metal devices. In this paper, a new method of fabricating a layered auxiliary cathode is proposed to improve the thickness uniformity of a micro-electroforming layer. In order to analyze the general applicability of the proposed method, four basic microstructures, namely circular, square, regular triangular, and regular hexagonal were used to study the effect of a layered auxiliary cathode on thickness uniformity through simulation and experimentation. The simulation results showed that with the help of the proposed auxiliary cathode, the thickness nonuniformity of four microstructures should decrease due to the reduced edge effect of the current density. The experimental results showed that the thickness uniformity of four microstructures fabricated via the proposed method was improved by 190.63%, 116.74%, 80.43%, and 164.30% compared to that fabricated via the traditional method, respectively. Meanwhile, the micro-gear was fabricated and the nonuniformity was reduced by 101.15% using the proposed method.

## 1. Introduction

Micro-electroforming is an important technology for micro-metal devices, which combines lithography and electrodeposition, and has the advantages of high precision, a wide range of processing sizes, and mass production [1,2]. Therefore, it is particularly suitable for manufacturing micro-molds, micro-sensors and micro-actuators in microelectronic mechanic systems (MEMS) [3,4,5,6]. However, there is the problem of thickness nonuniformity in the micro-electroforming process [7,8], manifested as an inconsistency between the height of the central and edge regions of the electroforming layer, which can affect the performance and usage requirements of micro-devices. Generally, post-processing (lapping/polishing) is required to ensure dimensional accuracy and surface quality, which prolongs the manufacturing cycle and increases manufacturing costs.

Currently, there are several methods to improve uniformity, including optimizing electrodeposition process parameters [9], adding additives [10], using pulse or reverse-pulse currents [11], setting auxiliary cathodes [12,13,14], adding an insulating shield [15], using auxiliary anodes [16], ultrasonic electrodepositing [17,18] and megasonic agitation [1]. Using auxiliary cathodes is an effective way to decrease the edge effect of a current in the micro electroforming process. The distance between the auxiliary cathode and the electroformed surface and the structure of the auxiliary cathode are two key factors determining the effectiveness of the auxiliary cathode on thickness uniformity. The ring or frame was used as the auxiliary cathode, which was placed at some distance from the electroformed structure [3,12,13]. Mehdizadeh et al. revealed that the improvement of thickness uniformity decreases with an increase in the distance between the auxiliary cathode and the electroformed surface [12]. Zhao et al. studied a coplanar auxiliary cathode with a similar local microstructure to that of mold in the fabrication of metal microfluidic chip mold, and the results showed that the coplanar cathode had an effect on the current density distribution of the microfluidic chip mold [14].

In this paper, a method of fabricating a layered auxiliary cathode is proposed to improve the current density distribution of the electroformed structure, thereby improving the uniformity of the electroformed layer. Differently from this method of using a coplanar auxiliary cathode, the proposed method adds an auxiliary cathode above the photoresist mold of the microstructure, which requires the cathode and auxiliary cathode to be placed in different photoresist mold layers and considers both the horizontal and vertical distances between the auxiliary cathode and the electroformed surface. The auxiliary cathode has an annular contour that completely covers the microstructure and is the exact same as that of the microstructure, which provides a unified solution for the selection of an auxiliary cathode structure. In order to analyze the general applicability of the proposed method for determining thickness uniformity, simulation analysis and an experiment were conducted on the micro electroforming process of four basic microstructures, namely circular, square, regular triangular, and regular hexagonal ones. The micro gear is the key element of various micro-systems and devices [19], and one of its main engineering limitations is its nonuniform thickness [2]. Finally, a micro gear was fabricated using the traditional method and proposed method, further verifying that this method could improve the uniformity of the micro electroforming layer.

## 2. Simulation of Layered Auxiliary Cathode

### 2.1. The Structure of Layered Auxiliary Cathode

**Figure 1:**
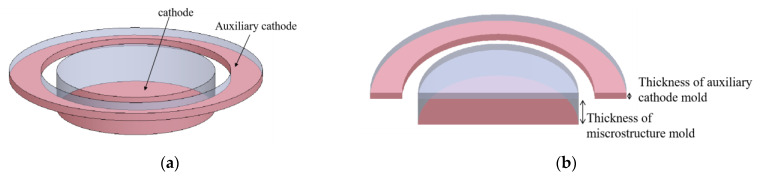
Schematic diagrams of layered auxiliary cathode illustrating a circular structure. (**a**) 3D schematic diagram; (**b**) Sectional 3D schematic diagram.

The layered auxiliary cathode is illustrated in schematic diagrams showing its circular structure in Figure 1. The entire cathode has two layers, including the microstructure cathode layer at the bottom and the auxiliary cathode at the top. The upper areas of the cathode and auxiliary cathode are grooves formed by photoresist and to be electroformed. The vertical distance between the two layers depends on the thickness of the underlying microstructure mold. The shape contour of the auxiliary cathode is the exact same as that of the external boundary of the microstructure, which allows it to achieve complete coverage of the microstructure. There is a certain distance between two layers of cathodes in the horizontal direction.

### 2.2. Geometric Model of Simulation

**Figure 2:**
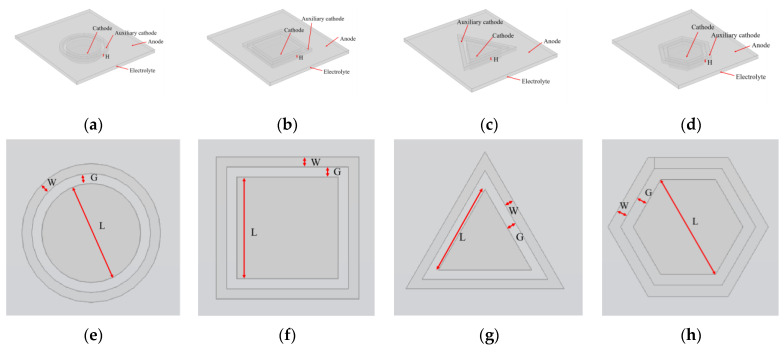
Geometric model and top view of four microstructures with auxiliary cathode. (**a**–**d**) geometric model of the circle, the square, the regular triangle and the regular hexagon, respectively; (**e**–**h**) top view of the circle, the square, the regular triangle and the regular hexagon, respectively.

The simulation is performed using COMSOL Multiphysics. Figure 2a–d presents the geometric models of four microstructures, which are a circle, the square, the regular triangle, and the regular hexagon, respectively, and the basic unit sizes (L) are all 100 µm. These microstructures include an arc, straight line, right angle, acute angle and obtuse angle, which cover the possible types of structures that may occur in micro-metal devices. The geometric models include the anode plane, the electrolyte domain, the cathode and auxiliary cathode. The anode is a Ni plate, so it is simplified as a plane. The cathode and auxiliary cathode are the electroforming surfaces. The sidewall is a photoresist vertical wall. The thickness of the microstructure mold is set as 20 µm and the time of electroforming is set as 180 s. Figure 2e–h presents a top view of four microstructures, where the gap between the microstructure cathode and the auxiliary cathode is abbreviated as G, the width of the auxiliary cathode is abbreviated as W, and the thickness of the auxiliary cathode mold is abbreviated as H. In order to compare the effect of the auxiliary cathode on thickness uniformity, G is set as 10 µm, 20 µm, 30 µm and 40 µm, W is set as 10 µm, 20 µm, 30 µm and 40 µm, and H is set as 5 µm, 10 µm, 15 µm and 20 µm. Nonuniformity, α, is used to quantify thickness uniformity of the electroformed layer. It is defined by

(1) $$\alpha =\frac{h_{max}-h_{min}}{h_{min}}\times 100\%$$

where, $h_{max}$ and $h_{min}$ are the maximum and minimum thickness of the electroformed layer, respectively.

### 2.3. Electroforming Model of Simulation

In the absence of the concentration gradients in the electrolyte, the electric field in the electrolyte can be described as [3,14,20]

(2) $$i_{l}=-\sigma \nabla \phi_{l}$$

(3) $${\nabla \cdot i}_{l}=0$$

where $i_{l}$ is local current density (A/m^2^), σ is the conductivity of the electrolyte (S/m) and $\phi_{l}$ is the electrolyte potential (V).

The Bulter–Volmer expression is used to describe the electrode reaction kinetics for the cathode and auxiliary cathode surfaces [3,14,20].

(4) $$i_{loc}=i_{0}\left(exp\left(\frac{a_{a}F\eta }{RT}\right)-exp\left(\frac{{-a}_{c}F\eta }{RT}\right)\right)$$

where $i_{loc}$ is local current density (A/m^2^) due to electrode reaction; $i_{0}$, $a_{a}$, $a_{c}$, F, R and T are the exchange density (A/m^2^), anode transfer coefficient, cathode transfer coefficient, Faraday constant (C/mol), universal gas constant (J/(mol·K)) and temperature (K), respectively. η is overpotential (V) and is defined by the following [3,14,20]:

(5) $$\eta =\phi_{s}-\phi_{l}-E_{eq}$$

where $\phi_{s}$ and $E_{eq}$ are the potential of the cathode surfaces (V) and equilibrium potential (V), respectively. The initial values of $\phi_{s}$ and η are both 0 V, so the initial condition for $\phi_{l}$ is as follows:

(6) $$\phi_{l}=-E_{eq}$$

The boundary condition of the total current is used for the electroforming area [3,14,20] and can be described as follows:

(7) $$I_{t}=-i_{avg}S$$

where $I_{t}$ is the total current (A), ‘−’ means that electrons outflow from the electrode, $i_{avg}$ is the average current density of the two cathodes (A/m^2^) and S is the total surface area of the electroforming layer (m^2^) which contains the surfaces of two cathodes.

Based on Faraday’s law, the nickel-depositing velocity can be described as follows [3,14,20]:

(8) $$V_{dep}=\frac{MN}{\rho }=-\frac{i_{loc}}{F}\frac{\gamma M}{n\rho }$$

where $V_{dep}$ is the depositing velocity (m/s), M is the molar mass of the nickel (kg/mol), γ is the stoichiometric coefficient, n is the electron number of the reaction and ρ is the density of the nickel (kg/m^3^). The simulation parameters are shown in Table 1.

### 2.4. Simulation Results and Discussion

**Figure 3:**
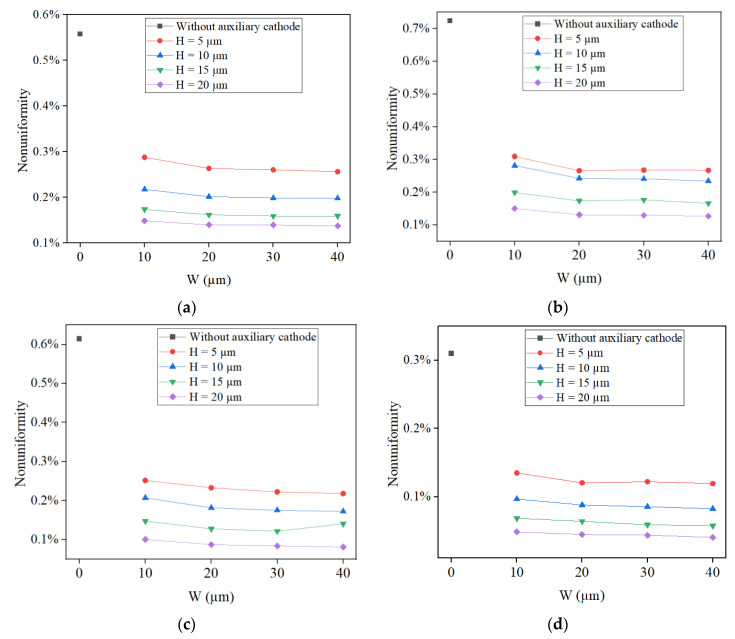
Calculated nonuniformity from simulation results of thickness distributions of four microstructures under different W and H conditions when G = 10 µm. (**a**) Nonuniformity of the circle; (**b**) nonuniformity of the square; (**c**) nonuniformity of the regular triangle; (**d**) nonuniformity of the regular hexagon.

Figure 3 shows the calculated nonuniformity from the simulation results of thickness distributions of four microstructures under different W (10 µm, 20 µm, 30 µm and 40 µm) and H (5 µm, 10 µm, 15 µm and 20 µm) conditions when G = 10 µm. As a comparison, the result without the auxiliary cathode (W = 0 µm) is also shown. From the calculated results, it can be seen that the nonuniformity trend of four microstructures is basically consistent, which is that as the layered auxiliary cathode is used in simulation, the uniformity of the electroformed layer significantly improves. At the same time, as the thickness of the auxiliary cathode mold increases, the nonuniformities of the electroformed layer become lower and lower. When W = 10 µm, the nonuniformities are the highest, while when W = 20 µm/30 µm/40 µm, the nonuniformities are basically the same. Therefore, when G = 10 µm, the optimal condition for H is 20 µm.

**Figure 4:**
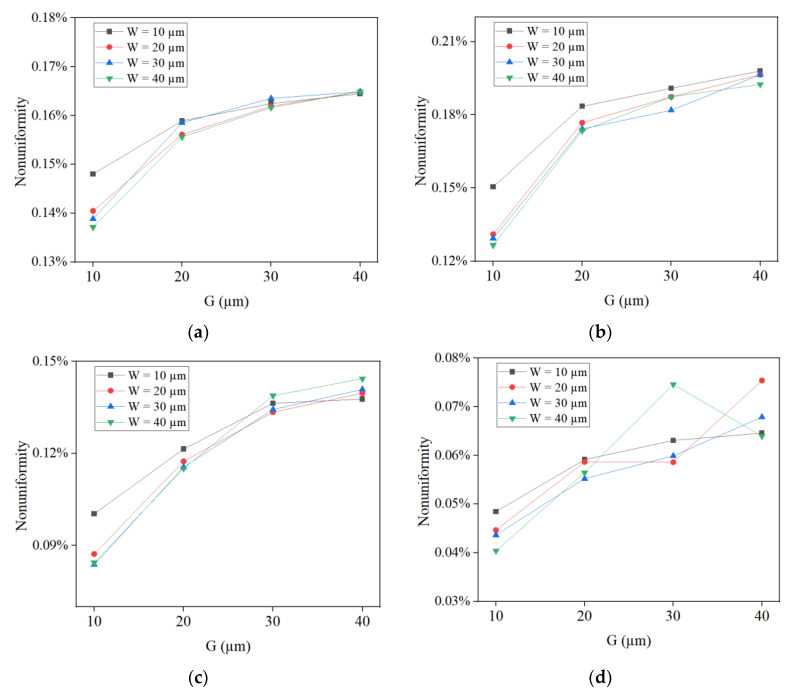
Calculated nonuniformity from simulation results of thickness distributions of four structures under different G and W conditions when H = 20 µm. (**a**) Nonuniformity of the circular structure; (**b**) nonuniformity of the square structure; (**c**) nonuniformity of the regular triangle structure; (**d**) nonuniformity of the regular hexagon structure.

Figure 4 shows the calculated nonuniformity from the simulation results of thickness distributions of the four structures under different G (10 µm, 20 µm, 30 µm and 40 µm) and W (10 µm, 20 µm, 30 µm and 40 µm) conditions when H = 20 µm, which is the optimal condition obtained from Figure 3. Under most conditions, the nonuniformity increases as G increases for four mcirostructures, so the optimal condition for G is 10 µm. When W = 10 µm, the nonuniformity is the highest. However, when W = 20 µm/30 µm/40 µm, the difference in nonuniformity is not significant. In order to reduce manufacturing difficulty, 40 µm is selected as the optimal condition for W. Therefore, based on the results of Figure 3 and Figure 4, the optimal conditions for H, G, and W are 20 µm, 10 µm, and 40 µm, respectively.

**Figure 5:**
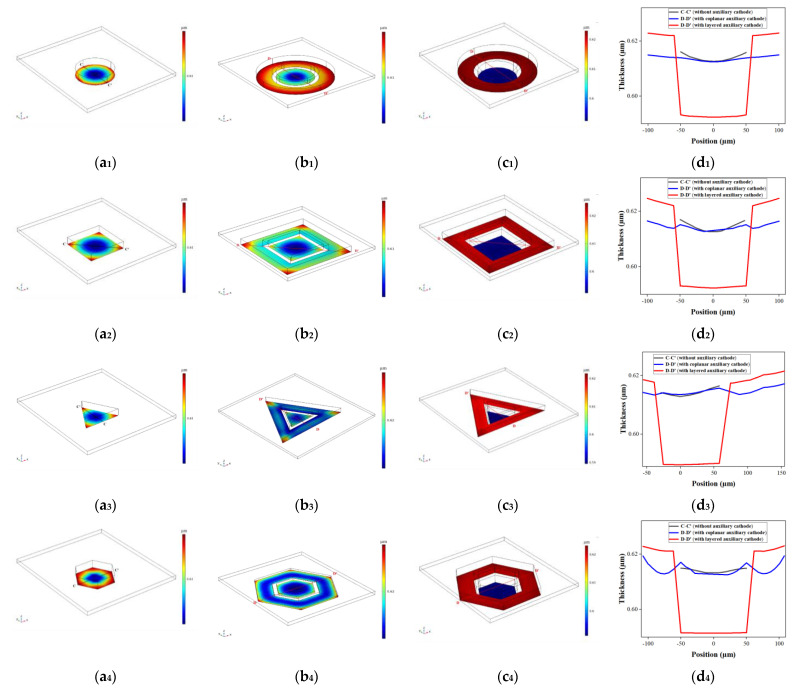
Simulation results of thickness distributions of four microstructures. (**a_1_**−**a_4_**) are the thickness distributions of the circular, square, regular triangular, and regular hexagonal microstructures without the auxiliary cathode, respectively; (**b_1_**−**b_4_**) are the thickness distributions of circular, square, regular triangular, and regular hexagonal microstructures with the coplanar auxiliary cathode under the conditions G = 10 µm and W =40 µm, respectively; (**c_1_**−**c_4_**) are thickness distributions of the circular, square, regular triangular, and regular hexagonal microstructures with the layered auxiliary cathode under the optimal conditions, respectively; (**d_1_**−**d_4_**) are thickness distributions of the circular, square, regular triangular, and regular hexagonal microstructures along C-C′ and D-D′, respectively.

Based on the optimal condition obtained above, the thickness distributions of four microstructures without the auxiliary cathode, with the coplanar auxiliary cathode and with the layered auxiliary cathode are shown in Figure 5(a_1_–a_4_), 5(b_1_–b_4_) and 5(c_1_–c_4_), respectively. Since the thickness of the sacrifice layer was about 2 µm [14], coplanar auxiliary cathode and cathode could be roughly in the same plane. The contoured structure of coplanar auxiliary cathode was arranged in the same way as that of layered auxiliary cathode, and the G and W conditions of the coplanar auxiliary cathode were set to 10 µm, and 40 µm, respectively. The thickness curves of four microstructures in dotted lines (C-C′ or D-D′) are shown in Figure 5(d_1_–d_4_). The difference between C-C′ and D-D′ is that D-D′ extends to the outer edge of the coplanar and layered auxiliary cathode. The results show that the use of coplanar and layered auxiliary cathodes both reduces the thickness, flattens the thickness distribution, and reduces the edge effect in the cathode structure region. Compared to coplanar assisted cathode, the layered assisted cathode greatly flattens the electroforming microstructures, where the electroforming thickness is lower. However, the electroforming thickness is higher in the area of auxiliary cathode, and gradually increases from the inner edge to the outer edge.

**Figure 6:**
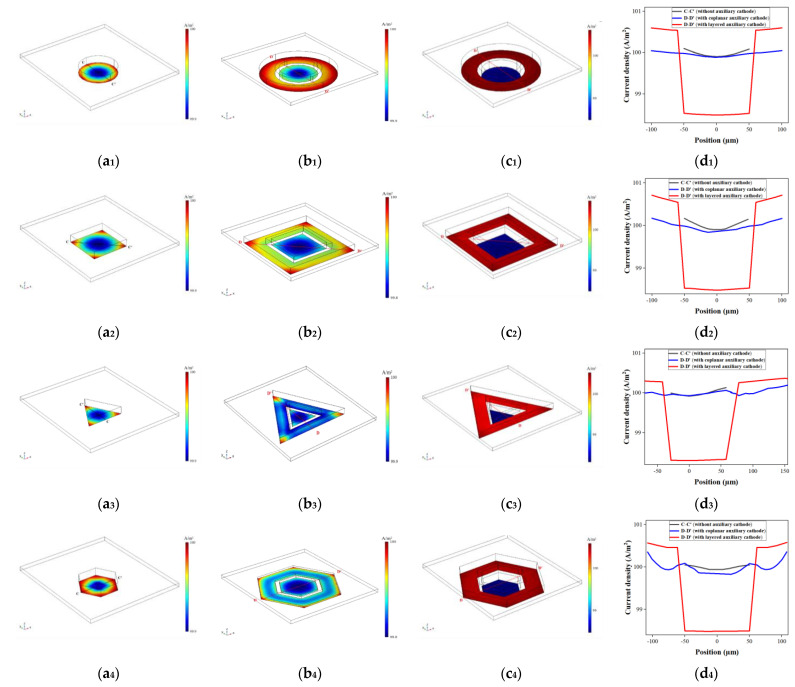
Simulation results of current density distributions of four microstructures. (**a_1_**−**a_4_**) are current density distributions of the circular, square, regular triangular, and regular hexagonal microstructures without an auxiliary cathode, respectively; (**b_1_**−**b_4_**) are current density distributions of the circular, square, regular triangular, and regular hexagonal microstructures with a coplanar auxiliary cathode under the conditions G = 10 µm and W = 40 µm, respectively; (**c_1_**−**c_4_**) are current density distributions of the circular, square, regular triangular, and regular hexagonal microstructures with a layered auxiliary cathode under optimal conditions, respectively; (**d_1_**−**d_4_**) are the current density distributions of the circular, square, regular triangular, and regular hexagonal microstructures along C-C′ or D-D′, respectively.

According to Faraday’s law, the thickness of the electroforming layer is proportional to the current density [14], so the current density distributions on the surface of these four microstructures can be used to analyze their thickness distributions. The current density distributions of four microstructures without the auxiliary cathode, with the coplanar auxiliary cathode and with the layered auxiliary cathode are showed in Figure 6(a_1_–a_4_), 6(b_1_–b_4_) and 6(c_1_–c_4_), respectively. Figure 6(d_1_–d_4_) shows the current density curves of the dotted lines (C–C′ or D–D′) of four microstructures simulated using three methods. Ming Zhao et al. explained that with the help of a coplanar auxiliary cathode, the current density is redistributed at the electroformed surface, while some current densities at the edge of mold’s surface are stolen by the auxiliary cathode [14]. However, the effect of a layered auxiliary cathode on stealing the current and decreasing the edge effect of the current is more obvious. One possible reason is that the change in the relative position of the cathode and auxiliary cathode causes a huge change in the redistribution of current density. Another possible reason is that the photoresist thickness at the cathode is higher than that without an auxiliary cathode and with a coplanar auxiliary cathode, which helps to reduce the edge effect of current. In our previous research, we found that as the thickness of the photoresist increased, the thickness nonuniformity of the electroformed gear structure decreased due to the reduced edge effect of the current density [21].

## 3. Experimental Details of Layered Auxiliary Cathode

### 3.1. Experimental Conditions

Electroforming equipment (Yamamoto-MS, A-52-ST6A-100B) was used to carry out the electroforming process. The composition of the electroforming solution was Ni[NH_2_SO_3_]_2_·4H_2_O (400 g·L^−1^), NiCl_2_ (20 g·L^−1^), H_3_BO_3_ (10 g·L^−1^) and a wetting agent (5 g·L^−1^). The operating temperature was 45 C, the pH value was about 4.0, and the current density was 1 A/dm^2^. The glass sputtered with a Cr/Au (10 nm/100 nm) seed layer was selected as the substrate. The four microstructures mentioned above and a micro gear which had a gear diameter of 960 µm, a tooth number of 10 and a modulus of 0.08 mm were experimented on. The condition parameters for H, G and Ware 20 µm, 10 µm and 40 µm as derived from THE simulation results, respectively.

### 3.2. Experimental Methods and Processes

**Figure 7:**
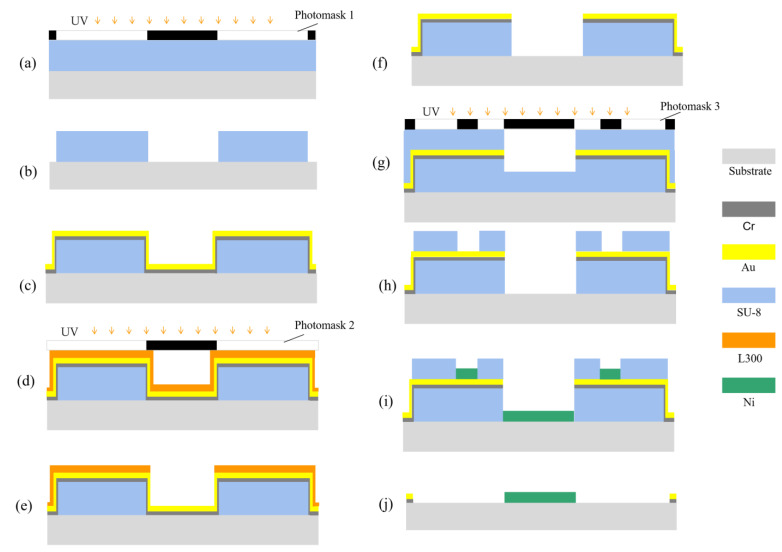
Processes of fabricating a microstructure with a layered auxiliary cathode. (**a**) Spin coating SU-8 and UV exposure; (**b**) development; (**c**) Cr/Au seed layer; (**d**) spin coating L300 and UV exposure; (**e**) development; (**f**) etching or slight-over etching of Cr/Au seed layer and removal of L300; (**g**) spin coating SU-8 and UV exposure; (**h**) development; (**i**) micro electroforming; (**j**) removal of SU-8 photoresist.

The fabrication processes of microstructures with a layered auxiliary cathode are shown in Figure 7. The fabrication details are as follows.

The process of fabricating a microstructure photoresist mold is shown in Figure 7a,b. A common negative photoresist (SU-8 2025) was spun at a pre-spin of 1000 rpm/10 s and a main spin of 3000 rpm/50 s. A soft bake (65 °C/60 s + 95 °C/6 min) on a contact hotplate was implemented. After soft baking, the resist was cooled down for 10 min to room temperature. Then, the sample was exposed to UV light at 6 mW/cm^2^ for 25 s using photomask 1, which contained the microstructures to be electroformed and conductive contact points at the edges. A post-exposure bake (65 °C/60 s + 95 °C/6 min) was carried out on a contact hotplate and let to cool down to room temperature. Then, the photoresist was developed in the SU-8 developer. After development, O_2_ plasma was used (100 W; 50 sccm; 60 s) to remove any remaining residue, and a microstructure photoresist mold of about 20 µm was obtained.

The process of fabricating an auxiliary cathode is shown in Figure 7c–h. In order to provide the conductive seed layer for the auxiliary cathode, the Cr/Au (5 nm/20 nm) seed layer was sputtered as shown in Figure 7c. However, sputtered Cr/Au appeared on the sidewall of the photoresist mold, which affected the electroforming of the microstructure. Some processes shown in Figure 7d–f were carried out to remove these redundant metals. A negative photoresist (DNR-L300-D1, L300) was spun at a pre-spin of 1000 rpm/10 s and a main spin of 3000 rpm/50 s. A soft bake (95 °C/60 s) on a contact hotplate was implemented. After soft baking, the resist was cooled down for 2 min to room temperature. Then, the sample was exposed to UV light at 6 mW/cm^2^ for 32 s using photomask 2, which contained the microstructures to be electroformed. A post-exposure bake (105 °C/90 s) was carried out on a contact hotplate and let to cool down to room temperature. Then, the photoresist was developed in the developer (AZ 300MIF). After development, O_2_ plasma was used (100 W; 50 sccm; 30 s) to remove any remaining residue, and Au(20 nm) and Cr(5 nm) were sequentially wet-etched or slightly over-etched which could ensure the complete removal of metals from the sidewall to eliminate the impact on electroforming, and then L300 was removed via soaking in acetone. Subsequently, the photoresist mold of the auxiliary cathode was fabricated as shown in Figure 7g,h. The detailed fabrication processes are the same as those in Figure 7a,b, except that photomask 3 replaced photomask 1 during the exposure process. Photomask 3 contained the microstructures to be electroformed, the structure of the auxiliary cathode and conductive contact points at the edges.

The micro electroforming and removal of the SU-8 photoresist are shown in Figure 7i,j. Micro electroforming for 30 min was performed, and the SU-8 photoresist was stripped in the SU-8 remover at 80 °C for 60 min to obtain the micro structure. Optionally, O_2_ plasma could be used (110 W; 50 sccm; 2 min) to remove any remaining residue.

**Figure 8:**
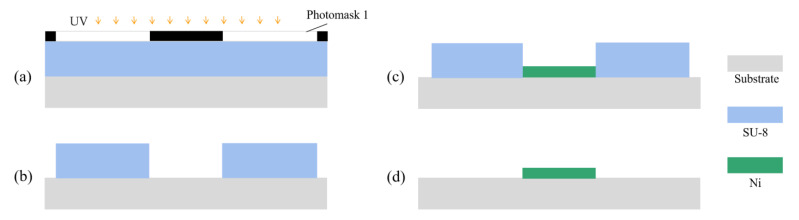
Processes of fabricating a microstructure without an auxiliary cathode. (**a**) Spin coating SU-8 and UV exposure; (**b**) development; (**c**) micro electroforming; (**d**) removal of SU-8 photoresist.

The fabrication processes of microstructures without an auxiliary cathode are shown in Figure 8 [22,23]. The fabrication details are as follows.

The processes of fabricating a microstructure mold are shown in Figure 8a,b, and are same as those in Figure 7a,b. The micro electroforming and removal of the SU-8 photoresist are shown in Figure 8c,d, and are carried out with the same process as that in as Figure 7i,j.

### 3.3. Measurements

The morphology was measured via field emission scanning electron microscopy (FE-SEM, Hitachi, S4800). The thickness distribution was measured using a laser scanning confocal microscope (LSCM, Olympus, Japan, OLS4000).

### 3.4. Experimental Results and Discussion

**Figure 9:**
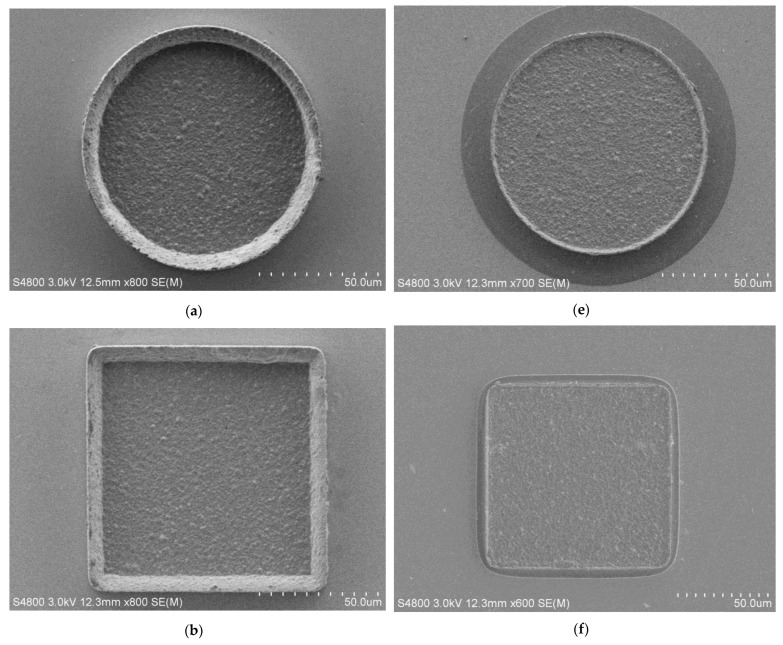

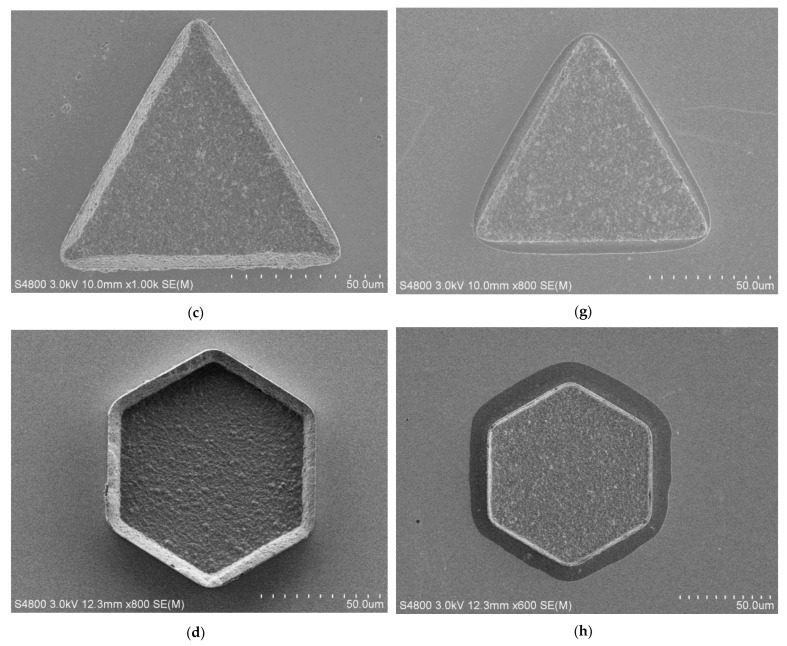
FE-SEM images of four microstructures fabricated via the traditional method and the proposed method. (**a**–**d**) are FE-SEM images of the circular, square, regular triangular, and regular hexagonal microstructures fabricated via the traditional method, respectively; (**e**–**h**) are the FE-SEM images of the circular, square, regular triangular, and regular hexagonal microstructures fabricated via the proposed method, respectively.

Figure 9 shows the FE-SEM photos of four microstructures fabricated via the traditional method and the proposed method. With the help of a layered auxiliary cathode, the bulges on the outer edge of these electroformed microstructures became less prominent. From the FE-SEM microphotographs of the proposed method, the peripheral pattern of the microstructure was that of a phenomenon of the slight over-etching of the substrate’s seed layer caused by the over-etching of the Cr/Au seed layer shown in Figure 7f, which indirectly verified that the Cr/Au on the side wall of the underlying mold in the area of microstructure had been completely removed, and eliminated its impact on the electroforming effect of microstructure edges.

**Figure 10:**
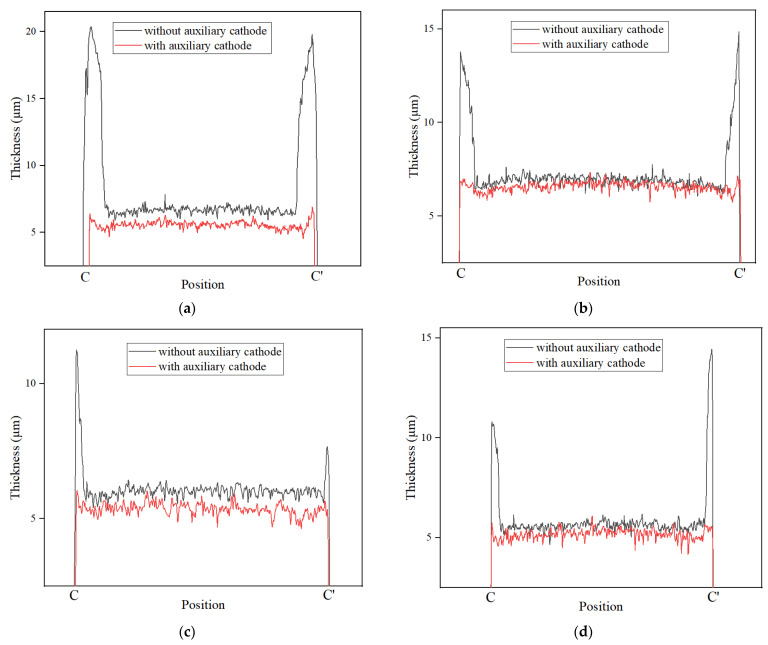
Dotted lines (C-C′) are the thickness distributions of four mcirostructures without and with a layered auxiliary cathode. (**a**) The thickness curves at the location (C-C′) of the circle; (**b**) the thickness curves at the location (C-C′) of the square; (**c**) the thickness curves at the location (C-C′) of the regular triangle; (**d**) the thickness curves at the location (C-C′) of the regular hexagon.

The thickness distributions of the dotted line (C-C′) of four microstructures were measured via LSCM, as shown in Figure 10. The position of C-C′ is the same as that in Figure 5 and Figure 6. It could be seen that the thickness of the edges of the microstructures fabricated via the proposed method is much lower than that of those fabricated via the traditional method, indicating that the proposed method could improve the edge effect of all electroformed microstructures. At the same time, the thickness with the auxiliary cathode was slightly lower than that without the auxiliary cathode, which was consistent with the results of the simulation of the current density distribution. The thickness nonuniformity is shown in Table 2. It was found that the nonuniformities of four microstructures were reduced by 190.63%, 116.74%, 80.43%, and 164.30% using the proposed method, respectively. Thus, the average nonuniformity was reduced by 138.03%, which indicated that regardless of the structure, the proposed method could effectively improve the uniformity of the micro electroforming layer.

**Figure 11:**
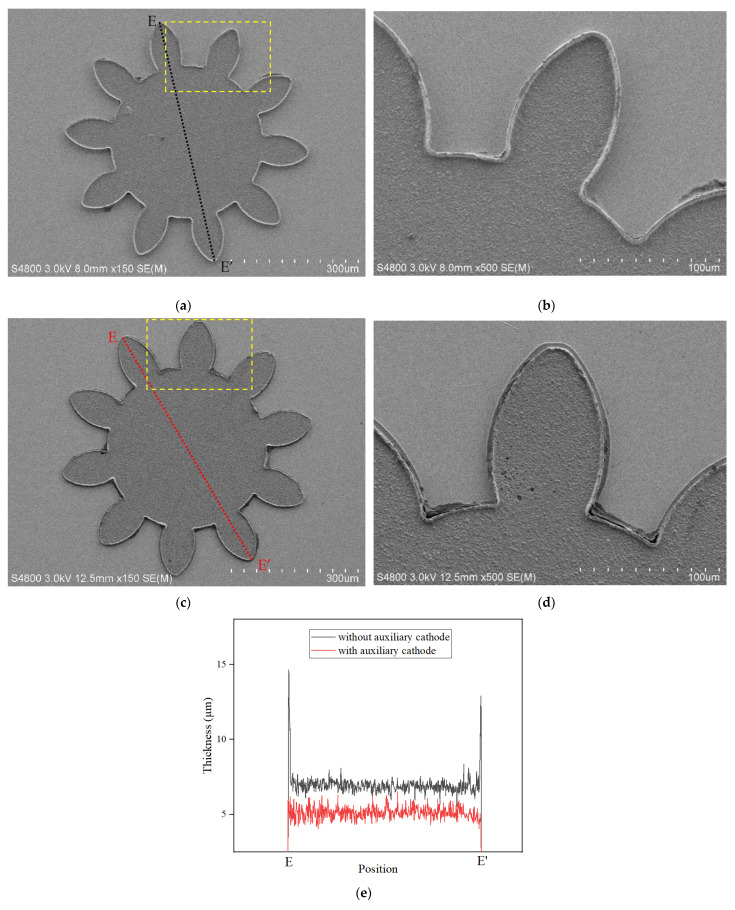
FE-SEM images and thickness distributions represented by dotted lines (E-E′) measured via LSCM on the micro gear without and with a layered auxiliary cathode. (**a**) FE-SEM image of the gear with the traditional method; (**b**) FE-SEM image of the local area of the gear located in the yellow-dashed box area in (**a**); (**c**) FE-SEM image of the gear in the proposed method; (**d**) FE-SEM image of the local area of the gear located in the yellow-dashed box area in (**c**); (**e**) the thickness distributions of dotted lines (E-E′) without and with the auxiliary cathode.

Figure 11a–d shows the FE-SEM photos of the micro gear fabricated using the traditional method and the proposed method. There were some residual fragments of the photoresist, which was caused by the incomplete removal of SU-8 from the micro gear structure, but these fragments did not affect the measurement of the thickness uniformity of the micro gear. If they were removed as thoroughly as possible by some means, such as ultrasound, the electroforming gear might have been damaged. These FE-SEM photos show that the bulges on the outer edge were improved due to the auxiliary cathode. The thickness distribution represented by the dotted line (E-E′) of the micro gear was measured via LSCM, as shown in Figure 11c. Due to the auxiliary cathode, the edge effect of the micro gear was significantly improved, which was consistent with the results of the four microstructures. The nonuniformity of thickness is shown in Table 3. It was found that the nonuniformity of the micro gear was reduced from 163.16% to 62.01% and was reduced by 101.15% due to the use of the proposed method, which indicated that the layered auxiliary cathode was a benefit in terms of improving the quality of the micro gear in the micro electroforming process.

## 4. Conclusions

In this paper, the method of fabricating a layered auxiliary cathode was proposed to improve thickness uniformity in the micro electroforming process. The effect of using an auxiliary cathode on the thickness uniformity of four basic microstructures was studied via simulation analysis. The simulation results show that the proposed method can reduce edge effects and improve the thickness uniformity of all microstructures. Compared to the four basic microstructures fabricated via the traditional method, the edge effect of those fabricated via the proposed method was smaller, and the nonuniformity was reduced by 190.63%, 116.74%, 80.43%, and 164.30%, which demonstrated the general applicability of the proposed method. Meanwhile, the nonuniformity of the fabricated micro gear was reduced by 101.15%, which further verified the effectiveness of the proposed method in terms of thickness uniformity. This method provides a new option for improving the thickness uniformity of micro electroforming metal structures.

## Figures and Tables

**Table 1:** Simulation parameters.

σ **(S/m)**	$i_{avg}$ **(A/m^2^)**	$a_{a}$	$a_{c}$	T(K)	$E_{eq}$	M	ρ	γ	n
0.95	100	1.5	0.5	318.15	−0.257	0.0586	8900	1	2

**Table 2:** Difference in α between four microstructures without and with auxiliary cathode.

	Method	Traditional (Without Auxiliary Cathode)	Proposed (With Auxiliary Cathode)
**Circle**	$h_{max}$ (µm)	20.353	6.906
	$h_{min}$ (µm)	5.936	4.536
	α	242.88%	52.25%
**square**	$h_{max}$ (µm)	14.852	7.332
	$h_{min}$ (µm)	6.073	5.737
	α	144.55%	27.81%
**regular triangular**	$h_{max}$ (µm)	11.24368	6.038
	$h_{min}$ (µm)	5.329	4.624
	α	111.01%	30.58%
**regular hexagonal**	$h_{max}$ (µm)	14.436	6.078
	$h_{min}$ (µm)	4.651	4.161
	α	210.38%	46.08%

**Table 3:** Difference in α between the micro gears without and with auxiliary cathode.

Method	Traditional (Without Auxiliary Cathode)	Proposed (With Auxiliary Cathode)
$h_{max}$ (µm)	14.635	6.513
$h_{min}$ (µm)	5.561	4.020
α	163.16%	62.01%

## Data Availability

Not applicable.
